# Automatic Detection of Atrial Fibrillation in ECG Using Co-Occurrence Patterns of Dynamic Symbol Assignment and Machine Learning

**DOI:** 10.3390/s21103542

**Published:** 2021-05-19

**Authors:** Nagarajan Ganapathy, Diana Baumgärtel, Thomas M. Deserno

**Affiliations:** Peter L. Reichertz Institute for Medical Informatics of TU Braunschweig and Hannover Medical School, Braunschweig, 38106 Lower Saxony, Germany; diana.baumgaertel@web.de (D.B.); thomas.deserno@plri.de (T.M.D.)

**Keywords:** electrocardiography, paroxysmal atrial fibrillation, RR intervals, symbolic pattern, classification, machine learning

## Abstract

Early detection of atrial fibrillation from electrocardiography (ECG) plays a vital role in the timely prevention and diagnosis of cardiovascular diseases. Various algorithms have been proposed; however, they are lacking in considering varied-length signals, morphological transitions, and abnormalities over long-term recordings. We propose dynamic symbolic assignment (DSA) to differentiate a normal sinus rhythm (SR) from paroxysmal atrial fibrillation (PAF). We use ECG signals and their interbeat (RR) intervals from two public databases namely, AF Prediction Challenge Database (AFPDB) and AF Termination Challenge Database (AFTDB). We transform RR intervals into a symbolic representation and compute co-occurrence matrices. The DSA feature is extracted using varied symbol-length V, word-size W, and applied to five machine learning algorithms for classification. We test five hypotheses: (i) DSA captures the dynamics of the series, (ii) DSA is a reliable technique for various databases, (iii) optimal parameters improve DSA’s performance, (iv) DSA is consistent for variable signal lengths, and (v) DSA supports cross-data analysis. Our method captures the transition patterns of the RR intervals. The DSA feature exhibit a statistically significant difference in SR and PAF conditions (*p* < 0.005). The DSA feature with W=3 and $\left|V\right|=3$ yield maximum performance. In terms of F-measure (F), rotation forest and ensemble learning classifier are the most accurate for AFPDB (F = 94.6%) and AFTDB (F = 99.8%). Our method is effective for short-length signals and supports cross-data analysis. The DSA is capable of capturing the dynamics of varied-lengths ECG signals. Particularly, the optimal parameters-based DSA feature and ensemble learning could help to detect PAF in long-term ECG signals. Our method maps time series into a symbolic representation and identifies abnormalities in noisy, varied-length, and pathological ECG signals.

## 1. Introduction

Cardiovascular diseases (CVDs) are the primary cause of death worldwide, with 45% in the European Union [1,2]. According to the World Health Organization (WHO), the number of deaths from CVDs has increased by 34% since 2000 [2,3]. Atrial fibrillation (AF) is one of the most common CVDs that has affected 33.5 million individuals worldwide and may affect 17.9 million in Europe by 2050 [3].

Electrocardiography (ECG) is the preferred technique to record the electrical activity of the heart. It is a standard clinical tool to detect and diagnose CVDs [4,5,6]. In a classical approach, ECG signals are monitored over a short time, and abnormalities are detected by visual inspection. Automatic analysis of ECG requires reliable identification of fiducial points for accurate measurements [7,8,9]. Most of the existing methods can cope only with relatively noise-free signals and steady features from local waves such as QRS complex, P, T, and U-wave. Hence, robust methods for automated assessment are essential to improve accuracy and reduce the false detection rates of AF.

A timely AF diagnosis yields plenty of opportunities for early prevention and a better lifestyle. By its nature, the detection of paroxysmal AF (PAF) is particularly challenging. In short-time traces, PAF is likely to be missed [7,10]. Often, authors exploit the heart rate variability (HRV) computed from the ECG for PAF diagnostics [2,7]. HRV is also the most common output of wearables assessing the heart dynamics by the variation of interbeat (RR) intervals [11]. In recent years, advancements in wearable devices and unobtrusive ECG sensors provided many options for long-term monitoring and PAF diagnostics. Unobtrusive capacitive-coupled ECG (cECG) sensors integrated into chairs and beds in smart homes, or seats of smart cars, allow long-term continuous recordings of ECG [12,13]. However, ECG signals acquired with such unobtrusive sensors differ from gold standard ECG. In addition, sensors in smart textiles and portable ECG devices with nonstandard limb-lead position requires robust approach for abnormality detection. Most of the state-of-the-art techniques fail abruptly on such conditions and have limited capability in real-time applications [4,14].

Several methods have been proposed for R-wave detection [2]. The R-wave is the most prominent part of the QRS complex and yields the simplest measure to diagnose PAF. For a healthy individual, the RR interval is regular, indicating a non-PAF rhythm. However, not all irregularities are pathological. Irregular but repetitive patterns are also associated with non-PAF rhythms. Contrarily, an irregularity that does not occur in patterns may indicate arrhythmia and, especially, AF [15,16]. Various approaches have been proposed for automatic HRV measurement which include the mean, standard deviation, or the mean of the averaged standard deviation of RR intervals and the root mean square of successive differences [7,11]. More advanced methods use transforms, mathematical morphology, hybrid approaches, and neural networks [8,17]. However, these methods suffer from limitations due to short-time recording and do not account for the pattern transitions in long-term ECG signals.

The symbolization approach has been used extensively to represent signals in a symbol space (alphabet), and it has several applications for biomedical signals [18,19]. In long-term signals, it is used to determine the transition of patterns, for example, due to varying bias, and to alleviate the influence of more extended patterns, noise, and inter-patient variations. Recently, Nui et al. used symbolization to detect the supraventricular and ventricular ectopic beats in ECG signals [16]. Mahajan et al. used symbols to identify congestive heart failure from RR intervals [11]. In most cases, the ECG signals are transformed into symbol space by discretizing the signal amplitude [11,19]. Due to the fixed-level amplitude discretization, the existing symbolization techniques cannot cope with long-term bias variations, motion artifacts, and other disruptive patterns. Thus, it limits the applicability of existing symbolization on short-term signals.

In addition to the conventional features obtained from long-term ECG signals, features extracted from the symbol space provide more in-depth details about the heart dynamics [11,18,20]. Various classification algorithms have been employed in the literature to differentiate regular sinus rhythms (SR) from PAF [5,6,19,21]. Classifiers such as random forest (RF) and support vector machine (SVM) have been used to determine healthy signals and congestive heart failure. Nui et al. combine symbolic pattern and deep learning to discriminate various beats of the ECG signals. They suggested a symbol-embedding matrix for improved classification [16].

## 2. Materials and Methods

In this work, we attempt to classify PAF segments in RR interval data using a dynamic symbolic assignment (DSA). For this, we use ECG signals and their RR interval labels from publicly available online databases. We represent the RR intervals on the time axis rather than the ECG signals’ amplitudes symbolically using discretization and symbolization. We compute co-occurrence matrices for each symbolized sequence and extract DSA features from the respective matrix to differentiate PAF segments. We fed the features into conventional classifiers, namely k-nearest neighbor (kNN), SVM, RF, rotation forest (RoF), and ensemble learning (EL).

**Figure 1:**
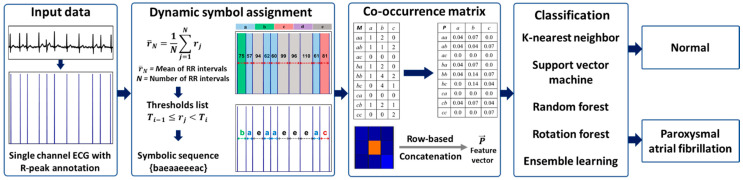
The overall pipeline of the proposed approach. The interbeat (RR) intervals are applied to dynamic symbol assignment (DSA) to map electrocardiography (ECG) signals to a symbolic sequence. The thresholds $T_{i}$ is used to maps the symbols in the RR interval. The pattern transition probability P is computed from co-occurrence pattern transition matrix M for symbols $\left\{a,b,c\right\}$. Finally, the P is transformed to a 1-dimensional array $\vec{P}$ using row-based concatenation, and the DSA features are extracted. The DSA features are fed to the k-nearest neighbor (kNN), support vector machine (SVM), random forest (RF), rotation forest (RoF), and ensemble learning (EL) classifiers to differentiate normal and paroxysmal atrial fibrillation (PAF) segments. The dark-blue arrow refers to the flow from one process to the next. The grey arrow refers to the intermediate outcome of the process.

We compare the performance of our DSA approach for different classifiers. In this study, the input data is obtained from online public databases, namely, AF Prediction Challenge Database (AFPDB) and AF Termination Challenge Database (AFTDB) [22,23,24]. The data is composed of ECG signals with normal SR and labeled PAF segments as ground truth. Our method is composed of three major steps: (1) DSA technique, (2) co-occurrence matrix, and (3) classification (Figure 1).

### 2.1. Dynamic Symbol Assignment (DSA)

**Figure 2:**
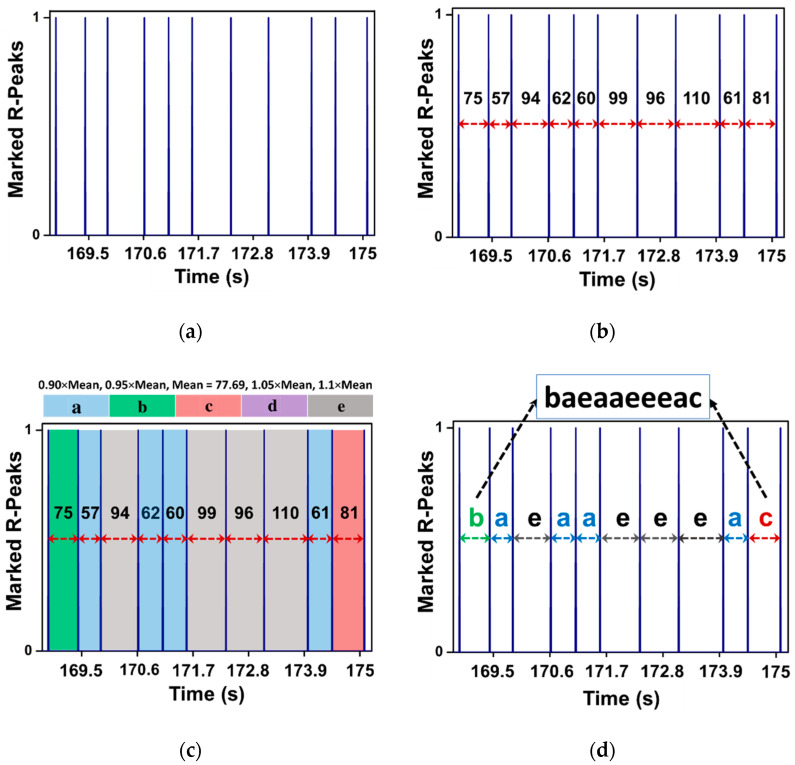
The pipeline of the dynamic symbol assignment (DSA) approach; representative RR intervals as input to the DSA method (**a**), distance evaluation of the input data (**b**), distance approximation using dynamic threshold lists (**c**), and representation of symbolic sequence after symbolization (**d**).

We first introduce our symbolization technique DSA (The source code of this study is available at: https://github.com/nagaganapathy/Dynamic_symbolic_Assignment.git (accessed on 1 May 2021)) designed explicitly for RR interval data. It is mainly used to determine the pattern transitions in the transformed symbolic sequences. The DSA approach consists of two major steps, namely, distance approximation, and symbolization. In most of the existing symbolization techniques, predefined thresholds are used as a discretization rule to map the sequential data into symbol sets [16,19]. The predefined thresholds may fail abruptly for time-series, as it requires prior domain knowledge for a specific application. To avoid the drawbacks of thresholds, we propose a dynamic thresholds list (Figure 2).

#### 2.1.1. Distance Approximation

**Figure 3:**
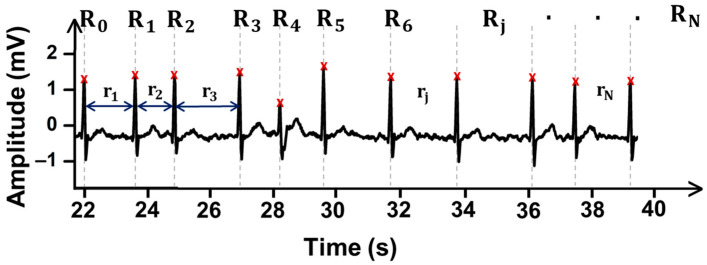
The electrocardiography (ECG) is composed of N+1 R-waves and N RR intervals.

At first, we determine the length of each RR interval:

(1) $$r_{j}=R_{j+1}-R_{j}$$

where $R_{j}$ denotes the $j^{th}$ R-wave in the ECG signal (Figure 3). The counter j is $j\;\epsilon \;{\mathbb{N}}_{0}$.

Let N+1 denote the number of R-waves in the ECG signal, then the mean distance ${\bar{r}}_{N}$ of all N intervals yields:

(2) $${\bar{r}}_{N}=\frac{1}{N}\sum_{j=1}^{N}r_{j}$$

Each $r_{j}$ is mapped onto a particular symbol $v_{i}\;\epsilon \;V$ of the vocabulary V, and its cardinality $\left|V\right|$ denotes the number of symbols in V. The number of symbols is initialized as the odd number to determine symmetric threshold lists for mapping $r_{j}$. For the mapping, we define $\left|V\right|-1$ threshold $T_{i}$. The thresholds $T_{i}$ are computed as:

(3) $$T_{i}={\bar{r}}_{N}\cdot \left(\left(1.00\pm t\right)\pm t\cdot k\right),\;k\in {\mathbb{N}}_{0},\;\mathrm{and}\;k<\frac{\left|V\right|-1}{2}$$

where $i\;\epsilon \;{\mathbb{N}}_{0}$, i≤2k, k depicts step size, and t is initialized as 0.05 for symmetrical threshold list using random search. For example, with $\left|V\right|=5$, the four thresholds ${T_{i}}^{5}\;\mathrm{for}\;\mathrm{mapping}\;\mathrm{are}\;0.90{\bar{r}}_{N},0.95{\bar{r}}_{N},1.05{\bar{r}}_{N},\;\mathrm{and}\;1.10{\bar{r}}_{N}$, for i=1,i=2,i=3, and i=4, respectively.

#### 2.1.2. Symbolization

After approximation and creating the dynamic thresholds lists, we use the corresponding symbol vocabulary $V=\left\{a,b,\ldots ,z\right\}$ to symbolize the elements of $r_{j}$. The thresholds list $T_{i}$ is used to symbolize the RR intervals and it is given by:

(4) $$\begin{array}{cc} D_{j}=v_{i}, & \mathrm{iff}\;T_{i-1}\leq r_{j}<T_{i} \end{array}$$

where $v_{i}\in V$ is the mapped symbol for the element $r_{j}$, $D=D_{1},D_{2},\ldots ,D_{j}$ is the final DSA symbolized sequence representation for $r_{j}$ elements, which is a symbolic sequence.

### 2.2. Co-Occurrence Patterns

Then, we use the DSA to determine the co-occurrence of the specific patterns of word size W, which is followed by a symbol. In this study, we chose W in the range of 2≤W≤5. For instance, in a symbolized sequence, a simple two-symbol $\left(W=2\right)$ pattern transition is where pattern ‘ab’ followed by ‘c’ or three-symbol $\left(W=3\right)$ pattern transition can be a pattern where ‘abc’ is followed by ‘d’. The identification of specific patterns and their pattern transitions with a symbol can be used to determine abnormality. We count the frequency of such patterns to compute the pattern transition probabilities (Table 1).

Considering an example of symbolic sequence $D=\left\{aabcbbbcbcbbbababc\right\}$ defined using $\left|V\right|=3$ with symbols $\left\{a,b,c\right\}$, we count the frequency of occurrence of pattern transition W=2, followed by a symbol in a lexico-logical order. Then, we normalize the matrices to determine pattern transition probability P and compare the characteristics of ECG signals with varying lengths. For the symbolic sequence D, the P is computed using the co-occurrence pattern transition matrix M, and it is mathematically expressed as:

(5) $$P=\frac{M_{i,j}}{\sum_{i}\sum_{j}M_{i,j}}$$

where $M_{i,j}$ is the co-occurrence matrix representing the frequency of patterns that occurred in the symbolic sequence D with $i^{th}$ row depicting the symbol pattern and $j^{th}$ column representing a symbol [11]. We computed the pattern transition probabilities similar to Akbilgic et al. [17] (Table 1). The selection of optimal W and $\left|V\right|$ is essential to determine the vital information necessary to identify disease conditions and to maintain low computational complexity.

Using our DSA method, the RR intervals of ECG are transformed into symbolic sequences. Later, P are computed for the derived symbolic sequence. Each P represents the pattern transition behavior of the signal segments. Finally, the P matrix is transformed into a single-row feature vector using row-based concatenation. For example, with W=3 and $\left|V\right|=3$, the single array $\vec{P}$ for symbolic sequence D is given as {$P_{1},P_{2},\ldots P_{i\cdot j},\ldots ,P_{27}\}$ where i and j are the rows and columns of the co-occurrence matrix. Likewise, the transition behavior of the patterns in the series for varied W and $\left|V\right|$ is computed to determine the probabilistic model of the series.

#### DSA Features

We extract DSA features, namely, maximum co-occurrence value with optimal values of W and $\left|V\right|$, from the symbolic sequence to discriminate SR and PAF segments. For this, we discretize the data using varied $\left|V\right|$ and calculate the maximum co-occurrence value from the single array $\vec{P}$ for varied $\left|V\right|$. In this study, we vary the $\left|V\right|$ as 3, 5, 7, 9 and the W as 2, 3, 4, 5, to determine the pattern transition behaviors, respectively. We employ the non-parametric Wilcoxon test on the DSA feature to determine the optimal values of W and $\left|V\right|$ that yield the maximum statistical significance.

### 2.3. Supervised Learning Classification

In the third step, we feed the feature to the supervised learning classifiers, namely, kNN, SVM, RF, RoF, and EL. Further, the grid search cross-validation technique is applied to tune the optimal parameters for all the classifiers mentioned above to ensure fair and unbiased comparison (Table 2) [25].

#### 2.3.1. SVM

SVM is one of the most preferred supervised learning methods for classification, regression, and prediction. The classifier differentiates the members of the classes by using the hyperplanes. It nonlinearly maps the input data into a higher-dimensional space. An optimal hyperplane maximizes the distance between the two different classes in training. For our data, we use a linear kernel function [26,27].

#### 2.3.2. kNN

It is a simple and popular classification technique that is based on clustering [26]. kNN classifier determines the label of test data by identifying the nearest groups of c clusters in the training set as its neighbors. It estimates the label of test data in three stages: (1) initialization of the training dataset as kNN, (2) computation of the distance metrics between the neighbors, and (3) classification of a test set based on the majority of labels of neighboring sets in the training set. Based on the classification accuracy, we set c=5.

#### 2.3.3. RF

It is a powerful ensemble learning-based classifier that computes the final decision based on the predicted outcome of the majority of the decision trees. These multiple trees are distinct and trained with data subsets grouped using random extraction with replacement [27]. As a result, some data samples are used more than once for training, which enhances the stability and reliability of the classifier for slight variations [28]. The average of the multiple decision trees is considered as the best results.

#### 2.3.4. RoF

The RoF is another ensemble learning-based approach. The features are divided into subsets and the principal components of each subset are applied to the decision trees [27]. Further, an equal number of rotations are performed on the feature subsets to generate new decision-making features. Additionally, these new features are also applied to decision trees to predict the labels of test data. Thus, RoF provides a more diverse and robust performance than the simple RF approach.

#### 2.3.5. EL

Adaboost EL improves accuracy by combining multiple strong and weak classifiers. It yields multiple models of classifiers for the same training set. Further, the best decision is computed by using majority voting labels. The learning technique considers the decision of multiple experts of different weights and thereby further improves the classification accuracy. We consider SVM, kNN, RF and RoF as weak classifiers for integrated output [29].

## 3. Evaluation

This section describes the hypotheses, experiments to prove (or to disapprove) the hypothesis, our validation approaches, and the performance matrices.

### 3.1. Hypotheses

All the experiments are designed to evaluate particular assumptions. We formulate five hypotheses:

**Hypotheses** **1** **(H1).** *DSA captures the dynamics of the time-series and learns the pattern transition for PAF conditions;*

**Hypotheses** **2** **(H2).** *DSA is a reliable technique to differentiate SR and PAF segments in various databases;*

**Hypotheses** **3** **(H3).** *There is an improvement in performance with optimal DSA parameters;*

**Hypotheses** **4** **(H4).** *DSA is consistent for a varied length of signals and does not require the same timestamp for comparisons;*

**Hypotheses** **5** **(H5).** *DSA supports cross-data analysis and is reliable for series of a reduced length.*

### 3.2. Experiments

All the experiments are designed to evaluate particular assumptions.

1. E1: The heatmaps represent the global transition patterns of SR and PAF segments. The pattern transition between SR and PAF segments is computed, and the obtained patterns are compared visually between the segments. H1 will be accepted if the heatmaps for SR and PAF segments are distinct and exhibit varying patterns.
2. E2: The DSA approach is applied to the various annotated databases, and the performance of the classifiers to differentiate SR and PAF segments is compared using their F-measure (F). H2 will be accepted if the F is higher (>90.0%).
3. E3: The average performance of DSA feature for different $\left|V\right|$ and W is compared individually using two databases. Statistical analysis is performed for varied W, $\left|V\right|$, and also to obtain comparable results. The performance of the classifiers is compared using the receiver operating characteristics (ROC) curve for multiple databases. The average values of the true positive rate and false positive rate obtained from each cross-validation are used. H3 is accepted if the performance gains for optimal parameters of $\left|V\right|$ and W. and the area under ROC curves (AUCs), are above 90.0% for multiple databases. Based on the higher values of F, the optimal parameters of $\left|V\right|$ and W are fixed for further experiments (E4 and E5).
4. E4: The average F and AUCs are computed on the DSA features for a varied length of the signals and the values are compared. Most of the authors have evaluated their methods on the same length of signals. Here, the DSA approach is evaluated for a varied signal length. H4 is accepted if varied signal lengths yield similar or improved performance.
5. E5: The F is computed on the series of two different but comparable databases to validate the cross-data analysis, and the values are compared. The majority of previous studies evaluated their methods on the same dataset [21,30,31,32]. Here, the one-minute PAF segments are obtained from AFTDB, and one-minute SR segments are extracted from AFPDB. We applied our DSA method across datasets. H5 is accepted if the F is above 95.0%.

### 3.3. Validation

We use 5-fold cross-validation [10,25,33]. To ensure reliable results, the 5-fold cross-validation is repeated ten times with randomly selected groups, and the average values are used for the performance comparison of the different classifiers. Furthermore, on the extracted features, statistical assessment is performed using a non-parametric Wilcoxon test with paired samples [34]. The average classification accuracy (ACC) of the results is defined as the performance of the classifier. We apply performance metrics, namely, precision (P), recall (R), F, and AUCs [34,35]. The P is defined as the number of correctly predicted PAF in the total number of predicted PAF. The R is defined as the index to determine the correctly predicted PAF to the total number of PAF. The F is given by the harmonic mean of P and R [2,34].

### 3.4. Databases

Several PAF databases are available online. Our selection criteria include the number of subjects, duration of recordings, the grade of other pathological conditions in the signals, and sampling rate (Table 3).

**Figure 4:**
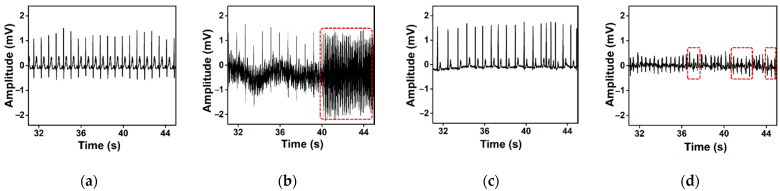
Representative ECG measurements of SR (**a**,**c**), and PAF (**b**,**d**) segments obtained from AFPDB and AFTDB, respectively. The identification of R-Peaks in PAF segments (within the red-rectangle area) are not easily identifiable.

The AF Prediction Challenge Database (AFPDB) has been used extensively in PAF-related studies [22,24]. Hence, we use it to validate experiments H1–H4. It contains 50 two-lead ambulatory ECG recordings, each includes 25- and 5-min length ECG segments that are obtained from 48 subjects. The records have been sampled at 128 Hz and digitized on a 16-bit resolution of over 200 A/D units per millivolt range (Table 2). Each recording contains a 25-min and a 5-min segment for normal rhythm and PAF, respectively (Figure 4a,b). It also includes another set of normal segments of 25- and 5-min lengths without any traces of PAF. A computer-aided method was used to annotate each beat.

The AF Termination Challenge Database (AFTDB) is also available online and accessible to the public. It contains 50 two lead ambulatory ECG recordings from 30 subjects [23]. The signals are sampled at 128 Hz with a resolution of 16 bits over a range of 200 A/D units per millivolt (Table 2). Each record contains one-minute length PAF extracted from the 20–24 h ambulatory ECG recording (Figure 4c,d). A computer-aided system has annotated the data individually. Of 50 recordings, 30 recordings are part of the training set in the challenge database and freely available. Thus, we considered only these 30 recordings for experiments.

In this study, the RR interval extracted from the annotated ECG signals of AFPDB and AFTDB databases is considered. Moreover, in the AFPDB dataset, the 5-min PAF segments and the first 5-min SR segments extracted from the 25-min long ECG signals are used for experiments H1–H4. Further, the 1-min PAF segments of the AFTDB dataset and the first one-minute SR segments of the 25-min-long ECG of the AFPDB dataset are is used for the experiments H2, H3, and H5.

## 4. Results

**Figure 5:**
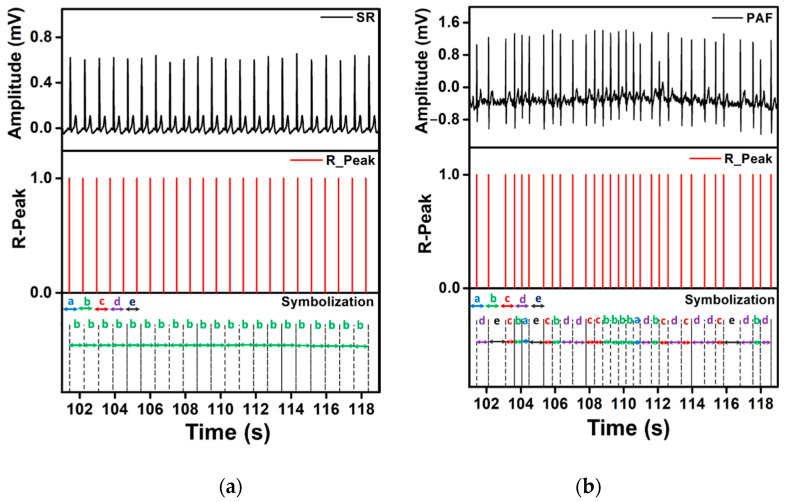
Representative SR (**a**), and PAF (**b**) segments with their corresponding RR interval and discretized symbolic sequences.

1. R1: The representative ECG signals, their corresponding RR interval, and the discretized series with symbolic sequences are shown in Figure 5. The differences in the distance between the R-spikes of the signals are visible in SR and PAF segments. The discretized symbolic sequence for SR segments contains relatively constant symbols, while the symbolic sequences of PAF segments are irregular and represent a frequent transition in patterns (Figure 5). For example, the symbol sequences $\left\{bbbbbbbbbbbbbbbbbbbb\right\}$ and $\left\{decbaecbddccbbbbadbcdcedbd\right\}$ are for SR and PAF segments, respectively.

**Figure 6:**
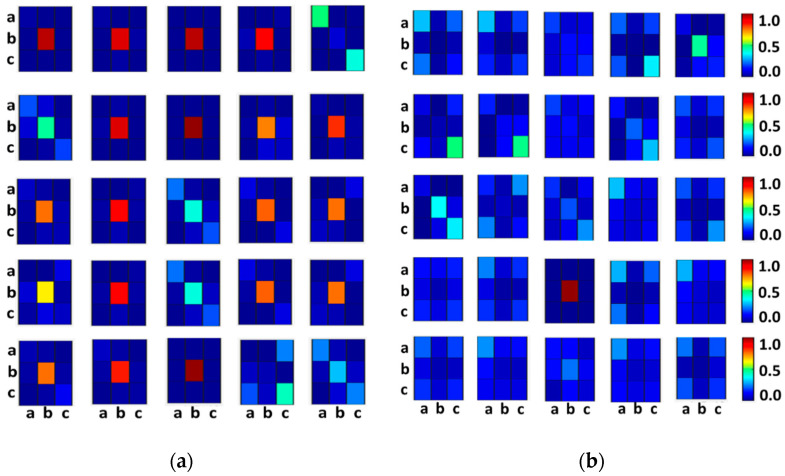
Representative heatmaps of transition patterns for SR (**a**), and PAF (**b**) segments using co-occurrence matrices.

The heatmaps for word size W=3 perform the best (Figure 6), which is in agreement with the literature [11,19]. The pattern transitions are consistent in SR but rather random in PAF segments. This is attributed to the fact that the lengths of RR intervals are regular in SR segments. However, for PAF segments, the lengths are inconsistent and hence, the pattern transition followed by symbol ‘b’ is found to be random. This approves our hypothesis H1.

**Figure 7:**
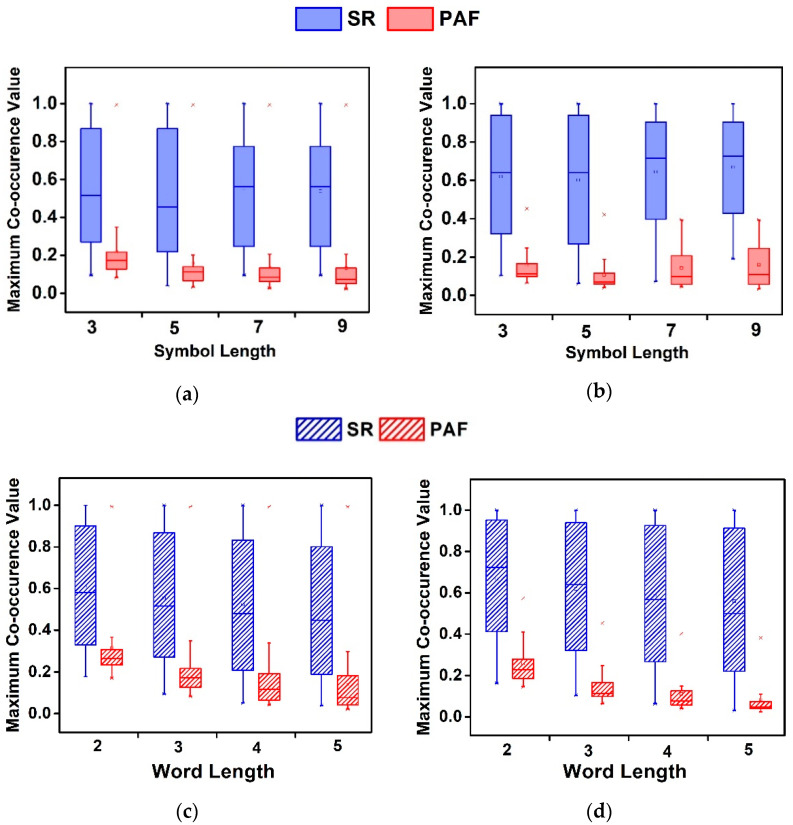
Boxplots representing the distribution of maximum co-occurrence values for varied $\left|V\right|$ with W=3 and for varied W with $\left|V\right|=3$ in AFPDB (**a**,**c**) and AFTDB (**b**,**d**).

2. R2: Comparing SR and PAF segments in both databases, the highest performance of F = 93.6% and F = 98.3% is obtained using W=4 and 3, respectively (Table 4). The RoF and EL classifiers have high performance for varied W. The P and R are also found to be the highest for W=3 in both databases. The RoF classifier yields the highest ACC of 93.6% and 98.3% for AFPDB and AFTDB databases, respectively. With varied classifiers, the DSA method differentiates SR and PAF segments in both databases, which confirms our hypothesis H2;
3. R3: The DSA features for varied $\left|V\right|$ discriminate SR and PAF segments in both databases (Figure 7). The maximum difference in the median in each database is 0.526 for $\left|V\right|=3$. The DSA feature for varied W with $\left|V\right|=3$ in AFPDB and is shown in Figure 7c,d. The higher value of the DSA feature indicates the presence of a similar pattern in the segments. The median is higher for SR segments and ranges from 0.5 to 0.75 for both databases. The mean decreases with higher values of W. The percentage difference of the average DSA feature is greater than 25% for varied W in both AFPDB and AFTDB databases. The smallest and largest difference in the average of DSA feature is observed with W=5 and 3 obtaining 0.122 and 0.526, respectively.

**Figure 8:**
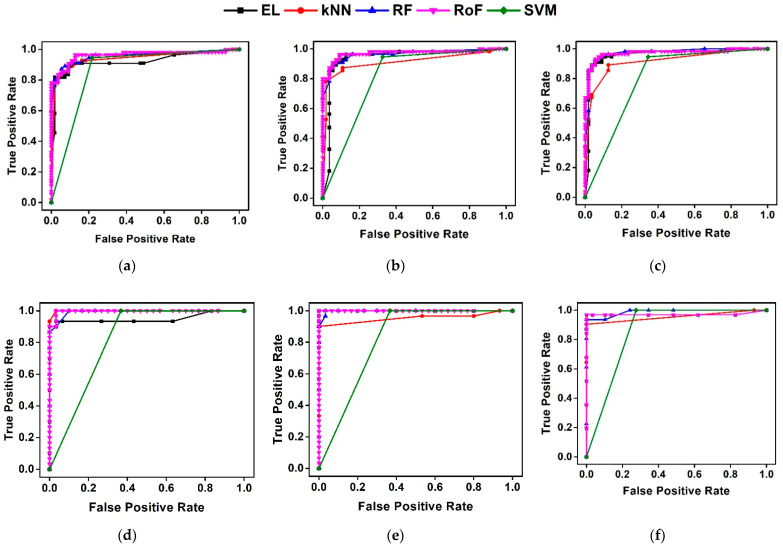
The average receiver operating characteristic (ROC) plots represent the performance of the classifiers for the DSA feature obtained using $\left|V\right|=3,\;5,\;7$ with W=3 for AFPDB (**a**–**c**) and AFTDB (**d**–**f**).

For AFPDB, the top three parameters: $\left|V\right|=9,\;7$, and 5 with W=3 obtained F of 94.6%, 92.7%, and 91.8 %, respectively. Similarly, for the AFTDB database, the top three parameters $\left|V\right|=5,\;7,$ and 3 with W=3 yield F of 99.8%, 98.3%, and 98.3%, respectively. When the word size W is set to 3 in both databases, the F is the highest (Table 5). Moreover, the DSA feature computed using $\left|V\right|$ and W as 3 exhibits a statistically significant difference in determining SR and PAF segments in both databases (*p* < 0.005). SVM and kNN perform unreliably for varied S in both databases. EL and RoF yield the highest AUC for varied V (Figure 8). The DSA feature has a discriminative power to identify PAF segments. Based on the F, $\left|V\right|$ and W are 3 as the optimal values for further experiments. Furthermore, this confirms our hypothesis H3.

**Figure 9:**
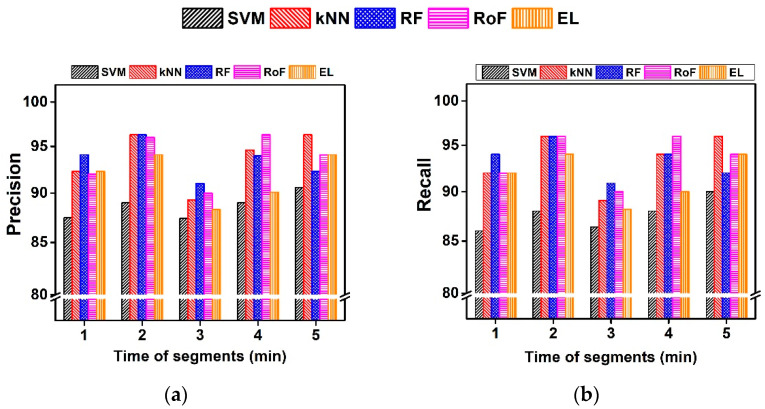
Comparison of P (**a**) and R (**b**) obtained for a varied length of time series by the DSA method and its classification using different classifiers.

4. R4: For the AFPDB database, kNN and RoF obtained the highest F of 96.2% and 96.0%, respectively (Table 6). However, the AUC is high for longer time series with 3-min and 4-min sequences (96.9% and 97.2% in Table 6). The gain in AUC for a longer sequence can be explained by the fact that longer signals characterize the dynamics of signals effectively. In terms of P and R, kNN and RoF are found to be consistently high for a varied length of the signals (Figure 9). Furthermore, the ACC is observed to be high and consistent for kNN, RF, and RoF in 3-min sequences. However, the pattern transition in varied lengths of the sequences is captured by our DSA method (Table 6), which confirms our hypothesis H4.
5. R5: The performance ranges from 84.1% to 99.8% for varied V using different classifiers. For the cross-dataset, the top three symbol lengths $\left|V\right|=5,\;7,$ and 9 obtained the highest F of 99.8% (Table 7). RoF and EL yield the highest performance. Except for SVM and kNN, the performance of DSA with varied $\left|V\right|$ is higher than 90.0%. Similarly, the AUC also ranges from 85.0% to 99.8% for varied S using different classifiers. The F is found to be consistent in $\left|V\right|=5\;\mathrm{and}\;7,$ for RoF and EL classifiers. The kNN classifier yields the lowest performance of classifiers. Thus, we can conclude that our method is effective in the cross-data analysis (H5 is true).

## 5. Discussion

### 5.1. Existing Challenges

The detection of PAF segments in ambulatory ECG recordings is essential and various works have been proposed. Many of these approaches are database-specific and cannot be applied to the signals with variable length [4,7,11,36]. Indeed, most of the PAF detection studies have been validated on an individual database with selected recordings to improve performance [2,10]. Recent advancements in recording devices such as long-term monitoring, wearables, and contactless ECG devices will improve continuous monitoring but will also yield more diverse quality and nature of signals [2,37]. Furthermore, domain adaptation and cross-data analysis in ECG signals have not been evaluated comprehensively to improve PAF detection and reduce false detection [38,39]. Therefore, the need for robust approaches with high performance in terms of PAF detection accuracy has been widely acknowledged [1,7,19].

Recently, symbolism techniques have been explored in physiological signals to characterize time-series dynamics and capture the transition pattern in pathological periods [16,20]. For instance, Akbilgic et al. [19] used symbolization to determine AF patterns in ECG signals. Mahajan et al. [11] applied symbols to discriminate normal and congestive heart failure conditions. Most of the symbolic approaches discretize the amplitude to captures the dynamic behavior of the signals [16,18,31]. However, this limits to selective leads and short-term recordings.

In this paper, we proposed a novel DSA method to symbolize RR intervals on the time axis to classify SR and PAF segments. To the best of our knowledge, our method is the first of its kind that uses a dynamic breakpoint list (Equation (4)), which overcomes the selective leads issue. Further, our implementation has been evaluated on multiple databases (Table 3). We have shown that DSA captures reliable transition patterns in sequences to discriminate SR and PAF in ECG with our experiments. DSA is robust, generalizable, and consistent (Figure 6).

### 5.2. Multi-Length ECGs

To evaluate the performance of the DSA method for a varied length of the sequences, we computed the F and AUCs for the signals of different lengths in the AFPDB database. The DSA method distinguishes the different segments on different lengths effectively. The highest F obtained by the DSA method is 99.8%. The top performance is achieved in sequences with a lower length (1 and 3 min). Except for SVM, the F of all other classifiers is above 90.0% across 1- to 5-min length signals. This indicates that the DSA method may detect PAF in mobile recordings, even with small traces.

It is observed that the DSA method has a clear tendency to discriminate SR and PAF segments using RR interval data, even in varied time segments. This is in line with the results reported by Mahajan et al., who concluded that the smaller segments of the signals are suitable for identifying PAF segments [30]. In most cases, the PAF episodes are asymptotic and episodic in nature, and hence, the diagnosis of PAF is quite challenging, especially in the early stages [22,23]. The DSA method captures the transition pattern of the sequences irrespective of signal length. Therefore, identifying irregular patterns in short sequences will aid in the timely diagnosis of AF segments.

### 5.3. Cross Dataset Analysis

Most of the studies have been restricted to a single database to identify and discriminate PAF segments. Park et al. [40] have verified their method on AFPDB and AFTDB databases to differentiate PAF segments. Although the technique has been tested on multiple databases, cross-data analysis and domain adaptation are rarely found to address the effectiveness of these routines in clinical conditions. Therefore, we tested the DSA method for cross-data analysis. In line with Natarajan et al. [41], the DSA method captures the transition patterns and discriminates the PAF segments in the cross-data analysis. Moreover, the performance of DSA increases for short segments in the cross-data analysis. Therefore, DSA is reliable for determining transition patterns and can be extended for other pathological conditions.

### 5.4. Application with Novel Sensing Devices

**Figure 10:**
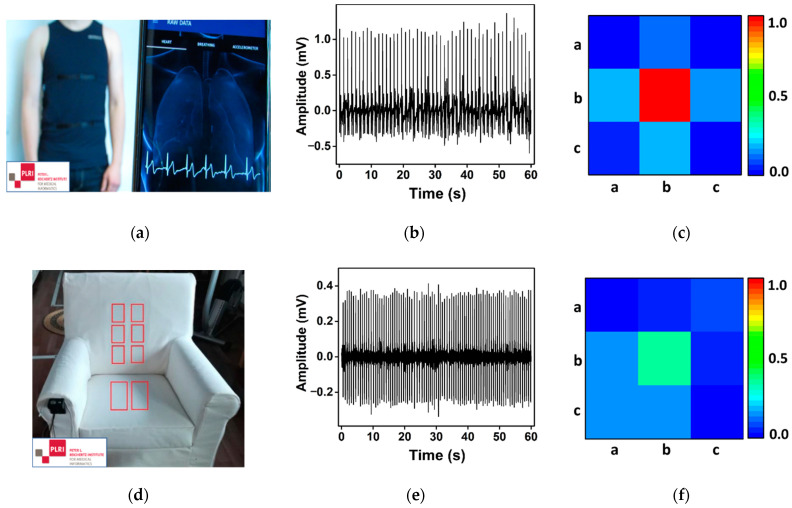
The wearable T-shirt (Pro-Kit, Hexoskin, Quebec, Canada) (**a**), the cECG chair (Smart Seat, Capical, Braunschweig, Germany) (**d**), the acquired one-minute ECG segments from these sensors (**b**,**e**), and the corresponding transition pattern evaluated using our method (**c**,**f**), respectively.

Although wearable sensor-based monitoring provides a continuous recording of physiological signals, the signals from wearables and capacitive sensors are noisy and may contain dropouts on several leads [14]. Furthermore, most of the existing techniques are not effective on such sensors due to motion artefacts and nonstandard ECG segments [14]. Therefore, we tested our method on the ECG segments acquired from sensors, namely, a wearable T-shirt (Pro-Kit, Hexoskin, QC, Canada) and the cECG chair (Smart Seat, Capical, Braunschweig, Germany) in real-time conditions (Figure 10) [42,43,44,45]. The STAPLE approach is used to determine the R peaks of acquired ECG segments [46]. In line with the proved hypothesis H1, our method also captures the transition patterns and determines the SR segments in such sensors (Figure 10). Moreover, our method also discriminates the SR segments in the nonstandard capacitive ECG segments. Therefore, our method is reliable for wearables and capacitive ECG sensors.

However, there is a limitation with the DSA approach. Although the performance of DSA-based features with the classification model is reliable to identify different disease conditions, the approach depends on the R-wave detection algorithms. Hence, the necessity of using a robust R-wave detection algorithm before the DSA symbolization algorithm is emphasized.

### 5.5. Comparison and Future Scope

The comprehensive evaluation of the DSA method has shown that our method can be extended to other leads and has higher potential. Further, the performance of DSA over state-of-the-art methods shows its effectiveness (Table 8). Except for Sutton et al., most of the methods used the split validation technique to evaluate their method. Further, Park et al. evaluated the performance of the method by combining both the databases as a single dataset. In the current study, multiple window length sequences have also been tested. The DSA method is computationally efficient, yielding only a few seconds (<9.0 s) for smaller symbol lengths to discriminate SR and PAF segments. Hence, it is possible to implement DSA-based PAF detection routines in PC or smartphones.

In the future, our method can be explored for signals from wearable devices, contactless ECGs, smart textiles, and other portable devices with nonstandard lead positions. In addition, RR interval sequences obtained from camera-based non-contact sensors can be used to evaluate the robustness of the DSA. Furthermore, DSA with deep learning could be explored to determine the abnormal transition patterns in the symbolic sequences.

## 6. Conclusions

In this paper, we have shown that the symbolization technique is a powerful tool for analyzing the irregularities and abnormalities of ECG signals. We proposed DSA to differentiate SR and PAF segments. For this, we use ECG signals and their RR intervals from AFPDB and AFTDB databases. We transform RR intervals into a symbolic representation and compute co-occurrence matrices. We extracted the DSA feature using varied $\left|V\right|$ and W; and then applied to five machine learning algorithms for classification. Our DSA method determines the transition patterns of the signals and robustly discriminates PAF segments in mobile-recorded ECG signals. Our symbolization approach with low $\left|V\right|$ and W parameters is effective to identify abnormalities. The combination of $\left|V\right|=3$ and W=3 features with an ensemble learning-based classifier gives the maximum performance (F = 94.0% and F = 99.80% for AFPDB and AFTDB database). Moreover, our method can differentiate PAF segments using variable-length signals and also supports cross-data analysis. Our method is amplitude-invariant and can be used for long-term signals. Our method is amplitude-invariant and can be used for long-term signals. Thus, it is useful for abnormalities detection in physiological signals. Furthermore, the approach can be explored for acoustics signals, mechanical signals, and other physiological signals.

## Figures and Tables

**Table 1:** An example of pattern transition probability P computed from co-occurrence pattern transition matrix M with word size W=3 for the symbolic sequence D.

Symbol Pattern	Pattern Transition Probabilities		
	a	b	c
‘aa’	0.04	0.07	0
‘ab’	0.04	0.04	0.07
‘ac’	0	0	0
‘ba’	0.04	0.07	0
‘bb’	0.04	0.14	0.07
‘bc’	0	0.14	0.04
‘ca’	0	0	0
‘cb’	0.04	0.07	0.04
‘cc’	0	0	0.07

**Table 2:** Optimal parameters for each classifier.

Database	Hyper-Parameters Range	Optimal
SVM	cost = (0.001, 0.01, 0.1, 1)	0.01
	gamma = (0.01, 0.1, 1)	0.1
	kernel = (Linear, Polynomial, Radial basis function)	Linear
kNN	c = (1, 2, 3…,10)	5
	weights = (Uniform, Distance)	Uniform
	metric = (Euclidean, Manhattan, Minkowski)	Euclidean
RF	max_depth = (10, 20, 30, …, 50, None)	None
	max_features = (‘auto’, ‘sqrt’)	sqrt
	min_samples_leaf = (1, 2, 3)	1
	min_samples_split = (2, 4, 6, 8, 10, 12)	10
	n_estimators = (100, 200, 300, …, 500)	100
RoF	max_features = (‘auto’, ‘sqrt’)	sqrt
	classifers = (‘RF’, ‘J48′, ’Decision tree’)	RF
	maxGroup = (1, 2, 3…, 10)	3
	minGroup = (1, 2, 3…, 10)	3
	projectionFilter = (‘PCA’, ’random’)	PCA
EL	number_of_classifers = (1,2,3…,10)	3
	classifiers_used = (SVM, kNN, RF, RoF)	SVM, RF, RoF

**Table 3:** Description of database in use.

Database	Leads	Subjects	Records	Sampling Rate (Hz)	Length (min)	Quantization Bit	AF Details (min)	Total Length (h)
PAF Prediction Challenge—2001	2	48	50	128	25, 5	16	5	24
AF Termination Challenge—2004	2	30	30	128	1	16	1	20–24

**Table 4:** Overall average performance (%) of the DSA method on the various databases using $\left|V\right|=7$ with varied W. Bold values indicate the best performance in a group.

W	Classifier		AFPDB				AFTDB		
		ACC	P	R	F	ACC	P	R	F
3	SVM	80.0	82.8	80.0	81.4	86.7	89.4	86.7	88.0
	kNN	81.8	84.8	81.8	83.3	81.7	86.7	81.7	84.1
	RF	91.8	91.9	91.8	91.8	93.5	96.9	96.7	96.8
	RoF	**92.7**	**92.8**	**92.7**	**92.7**	98.3	98.4	98.3	98.3
	EL	91.8	91.9	91.8	91.8	**98.3**	**98.4**	**98.3**	**98.3**
4	SVM	77.3	81.0	77.3	79.1	85.0	88.4	85.0	86.7
	kNN	52.7	61.4	52.7	56.7	50.0	50.0	50.0	50.0
	RF	81.8	84.0	81.8	82.9	93.3	94.1	93.3	93.7
	RoF	**93.6**	**93.7**	**93.6**	**93.6**	**98.3**	**98.4**	**98.3**	**98.3**
	EL	89.1	89.1	89.1	89.1	93.3	93.3	93.3	93.3
5	SVM	77.3	81.9	77.3	79.5	85.0	88.4	85.0	86.7
	kNN	50.0	50.0	50.0	50.0	50.0	50.0	50.0	50.0
	RF	72.7	82.4	72.7	77.2	48.3	83.5	75.0	79.0
	RoF	88.2	89.2	88.2	88.7	85.0	87.1	85.0	86.0
	EL	**91.8**	**91.9**	**91.8**	**91.8**	**95.0**	**95.0**	**95.0**	**95.0**

**Table 5:** The average F (%) for varied $\left|V\right|$ in two databases. Bold values indicate the best performance in a group.

W	Classifier	AFPDB				AFTDB			
		3	5	7	9	3	5	7	9
3	SVM	86.9	82.1	81.4	80.6	84.1	89.1	88.0	88.0
	kNN	89.2	89.0	83.3	72.5	98.3	84.1	84.1	62.8
	RF	**90.9**	**91.8**	91.8	92.0	93.7	96.7	96.8	88.9
	RoF	90.0	91.8	**92.7**	**94.6**	**98.3**	**99.8**	**98.3**	**98.3**
	EL	88.2	91.0	91.8	93.6	95.2	98.3	98.3	95.0
4	SVM	85.2	79.9	79.1	79.1	81.5	80.2	86.7	86.7
	kNN	90.1	62.8	56.7	50.0	92.3	50.0	50.0	62.8
	RF	**91.8**	91.1	82.9	84.8	95.0	88.1	93.7	85.4
	RoF	90.9	**92.7**	**93.6**	**93.6**	**96.8**	**99.8**	**98.3**	**96.8**
	EL	88.2	92.8	89.1	91.8	91.7	99.8	93.3	93.4
5	SVM	82.9	79.1	79.5	60.7	78.9	77.7	86.7	47.1
	kNN	73.7	60.7	50.0	50.0	63.9	50.0	50.0	62.8
	RF	87.5	77.9	77.2	67.8	95.0	73.9	79.0	70.4
	RoF	90.0	87.9	88.7	77.9	**99.4**	84.1	86.0	80.3
	EL	**92.7**	**93.6**	**91.8**	**85.5**	98.3	**95.0**	**95.0**	**81.7**

**Table 6:** The average F (%) and AUCs (%) obtained by the DSA method for a varied length of the time segments in the AFPDB database. Bold values indicate the best performance in a column.

Classifier	Time Segments (min)														
	1			2			3			4			5		
	ACC	F	AUCs	ACC	F	AUCs	ACC	F	AUCs	ACC	F	AUCs	ACC	F	AUCs
SVM	85.7	86.7	86.0	88.0	88.4	88.0	88.0	88.4	88.0	90.0	90.0	90.0	86.4	86.9	86.4
kNN	92.0	92.1	96.2	94.0	**94.2**	**96.1**	96.0	**96.1**	**96.9**	**96.0**	**96.2**	**97.2**	89.0	89.1	93.9
RF	**94.0**	**94.0**	**97.6**	94.0	94.0	96.1	96.0	96.1	95.8	92.0	92.1	96.1	**90.1**	**90.1**	95.8
RoF	92.0	92.0	97.1	**96.0**	96.1	95.5	**96.0**	96.0	96.0	94.0	94.1	95.7	90.0	90.0	**96.2**
EL	92.0	92.1	96.0	90.0	90.0	95.4	94.0	94.1	92.0	94.0	94.1	94.1	88.2	88.3	92.2

**Table 7:** The average F (%) and AUC (%) obtained by the DSA method for cross-dataset using W=3. The PAF and SR segments are obtained from AFTDB and AFPDB databases, respectively. Bold values indicate the best performance in a column.

Classifier	Symbol Lengths											
	3			5			7			9		
	ACC	F	AUCs	ACC	F	AUCs	ACC	F	AUCs	ACC	F	AUCs
SVM	85.0	86.7	85.0	85.0	86.7	85.0	85.0	86.7	85.0	85.00	86.7	85.0
kNN	95.0	95.1	99.3	88.3	89.4	94.0	82.7	84.1	95.4	55.0	63.9	65.7
RF	93.3	93.3	99.3	96.7	96.7	99.4	95.0	95.2	99.8	98.3	98.3	99.8
RoF	96.7	96.7	**99.6**	**99.8**	**99.8**	**99.8**	**99.8**	**99.8**	**99.8**	**99.8**	**99.8**	**99.8**
EL	**98.3**	**98.3**	96.9	99.8	99.8	99.8	99.8	99.8	99.8	96.7	96.8	99.8

**Table 8:** Comparison of the state-of-the-art methods with our approach. Bold values indicate the best performance in a column.

Existing Methods	Database	Classifiers	Validation	Length (min)	P	R	F	ACC
Mohebbi and Ghassemian [21]	AFPDB	SVM	Split	-	96.3	93.1	-	-
Zong et al. [30]	AFPDB	ARMA, FL	Split	30	-	-	-	80.88
Sutton et al. [31]	AFPDB	LD, LR, DT, RF	5-fold	1	**100**	73.6	-	82.0
Pourbabaee et al. [32]	AFPDB	CNN, kNN, SVM, MLP	Split	5	-	-	-	91.0
Park et al. [40]	Combined AFPDB and AFTDB	SVM	4-fold	1	91.4	92.9	-	-
Our Method	AFPDB	SVM, kNN, RF, RoF, EL	5-fold	1, 5	94.6	94.5	94.6	94.0
	AFTDB			1	99.8	**99.8**	**99.8**	**99.8**

## Data Availability

The ECG signals are obtained from the AFPDB and AFTDB database. All the databases are public domain database and available online: https://physionet.org/about/database/ (accessed on 1 November 2020). The source code of this study is available at https://github.com/nagaganapathy/Dynamic_symbolic_Assignment.git (accessed on 1 May 2021).

## References

[1] Morillo C.A., Banerjee A., Perel P., Wood D., Jouven X. (2017). Atrial fibrillation: The current epidemic. J. Geriatr. Cardiol..

[2] Kashif M., Jonas S.M., Deserno T.M. (2016). Deterioration of R-Wave Detection in Pathology and Noise: A Comprehensive Analysis Using Simultaneous Truth and Performance Level Estimation. IEEE Trans. Biomed. Eng..

[3] Kaptoge S., Pennells L., De Bacquer D., Cooney M.T., Kavousi M., Stevens G., Riley L.M., Savin S., Khan T., Altay S. (2019). World Health Organization cardiovascular disease risk charts: Revised models to estimate risk in 21 global regions. Lancet Glob. Health.

[4] Deserno T.M., Marx N. (2016). Computational electrocardiography: Revisiting Holter ECG monitoring. Methods Inf. Med..

[5] Chandra B.S., Sastry C.S., Jana S., Patidar S. Atrial fibrillation detection using convolutional neural networks. Proceedings of the 2017 Computing in Cardiology (CinC).

[6] He R., Wang K., Zhao N., Liu Y., Yuan Y., Li Q., Zhang H. (2018). Automatic Detection of Atrial Fibrillation Based on Continuous Wavelet Transform and 2D Convolutional Neural Networks. Front. Physiol..

[7] Rizwan A., Zoha A., Ben Mabrouk I., Sabbour H.M., Al-Sumaiti A.S., Alomainy A., Imran M.A., Abbasi Q.H. (2020). A Review on the State of the Art in Atrial Fibrillation Detection Enabled by Machine Learning. IEEE Rev. Biomed. Eng..

[8] Burguera A. (2018). Fast QRS Detection and ECG Compression Based on Signal Structural Analysis. IEEE J. Biomed. Health Inform..

[9] Spicher N., Kukuk M. (2020). Delineation of Electrocardiograms Using Multiscale Parameter Estimation. IEEE J. Biomed. Health Inform..

[10] Andersen R.S., Peimankar A., Puthusserypady S. (2019). A deep learning approach for real-time detection of atrial fibrillation. Expert Syst. Appl..

[11] Mahajan R., Viangteeravat T., Akbilgic O. (2017). Improved detection of congestive heart failure via probabilistic symbolic pattern recognition and heart rate variability metrics. Int. J. Med. Inform..

[12] Wang J., Warnecke J.M., Haghi M., Deserno T.M. (2020). Unobtrusive Health Monitoring in Private Spaces: The Smart Vehicle. Sensors.

[13] Castro I., Patel A., Deviaene M., Huysmans D., Borzee P., Buyse B., Testelmans D., Van Huffel S., Varon C., Torfs T. (2020). Unobtrusive, through-clothing ECG and Bioimpedance Monitoring in Sleep Apnea Patients. TC.

[14] Satija U., Ramkumar B., Manikandan M.S. (2018). A Review of Signal Processing Techniques for Electrocardiogram Signal Quality Assessment. IEEE Rev. Biomed. Eng..

[15] Yin H., Yang S., Zhu X., Ma S., Chen L. (2015). Symbolic representation based on trend features for biomedical data classification. Technol. Health Care.

[16] Niu J., Tang Y., Sun Z., Zhang W. (2019). Inter-Patient ECG Classification with Symbolic Representations and Multi-Perspective Convolutional Neural Networks. IEEE J. Biomed. Health Inform..

[17] Adam M., Oh S.L., Sudarshan V.K., Koh J.E., Hagiwara Y., Tan J.H., Tan R.S., Acharya U.R. (2018). Automated characterization of cardiovascular diseases using relative wavelet nonlinear features extracted from ECG signals. Comput. Methods Programs Biomed..

[18] Akbilgic O., Howe J.A. (2017). Symbolic pattern recognition for sequential data. Seq. Anal..

[19] Akbilgic O., Howe J.A., Davis R.L. (2016). Categorizing atrial fibrillation via symbolic pattern recognition. J. Med. Stat. Inform..

[20] Zhang C., Chen Y., Yin A., Wang X. (2019). Anomaly detection in ECG based on trend symbolic aggregate approximation. Math. Biosci. Eng..

[21] Mohebbi M., Ghassemian H. (2012). Prediction of paroxysmal atrial fibrillation based on non-linear analysis and spectrum and bispectrum features of the heart rate variability signal. Comput. Methods Programs Biomed..

[22] Moody G., Goldberger A., McClennen S., Swiryn S. (2001). Predicting the onset of paroxysmal atrial fibrillation: The computers in cardiology challenge 2001. Comput. Cardiol..

[23] Moody G. (2004). Spontaneous termination of atrial fibrillation: A challenge from physionet and computers in cardiology 2004. Comput. Cardiol..

[24] Goldberger A.L., Amaral L.A., Glass L., Hausdorff J.M., Ivanov P.C., Mark R.G., Mietus J.E., Moody G.B., Peng C.-K., Stanley H.E. (2000). Physiobank, physiotoolkit, and physionet: Components of a new research resource for complex physiologic signals. Circulation.

[25] Le N.Q.K., Do D.T., Hung T.N.K., Lam L.H.T., Huynh T.-T., Nguyen N.T.K. (2020). A Computational Framework Based on Ensemble Deep Neural Networks for Essential Genes Identification. Int. J. Mol. Sci..

[26] Venugopal G., Navaneethakrishna M., Ramakrishnan S. (2014). Extraction and analysis of multiple time window features associated with muscle fatigue conditions using sEMG signals. Expert Syst. Appl..

[27] Karthick P., Ghosh D.M., Ramakrishnan S. (2018). Surface electromyography based muscle fatigue detection using high-resolution time-frequency methods and machine learning algorithms. Comput. Methods Programs Biomed..

[28] Wu X., Gao Y., Jiao D. (2019). Multi-Label Classification Based on Random Forest Algorithm for Non-Intrusive Load Monitoring System. Processes.

[29] Huang Q., Chen Y., Liu L., Tao D., Li X. (2020). On Combining Biclustering Mining and AdaBoost for Breast Tumor Classification. IEEE Trans. Knowl. Data Eng..

[30] Zong W., Mukkamala R., Mark R.G. A methodology for predicting paroxysmal atrial fibrillation based on ECG arrhythmia feature analysis. Proceedings of the Computers in Cardiology 2001 Vol28 (Cat No01CH37287).

[31] Sutton J.R., Mahajan R., Akbilgic O., Kamaleswaran R. (2019). Physonline: An open source machine learning pipeline for real-time analysis of streaming physiological waveform. IEEE J. Biomed. Health Inform..

[32] Pourbabaee B., Roshtkhari M.J., Khorasani K. (2018). Deep convolutional neural networks and learning ECG features for screening paroxysmal atrial fibrillation patients. IEEE Trans. Syst. Man Cybern. Syst..

[33] Le N.Q.K., Do D.T., Chiu F.-Y., Yapp E.K.Y., Yeh H.-Y., Chen C.-Y. (2020). XGBoost Improves Classification of MGMT Promoter Methylation Status in IDH1 Wildtype Glioblastoma. J. Pers. Med..

[34] Ganapathy N., Veeranki Y.R., Swaminathan R. (2020). Convolutional neural network based emotion classification using electrodermal activity signals and time-frequency features. Expert Syst. Appl..

[35] Zhang Y., Guo Y., Yang P., Chen W., Lo B. (2019). Epilepsy Seizure Prediction on EEG Using Common Spatial Pattern and Convolutional Neural Network. IEEE J. Biomed. Health Inform..

[36] Habib A., Karmakar C., Yearwood J. (2020). Choosing a sampling frequency for ECG QRS detection using convolutional networks. arXiv.

[37] Castro I.D., Varon C., Torfs T., Van Huffel S., Puers R., Van Hoof C. (2018). Evaluation of a Multichannel Non-Contact ECG System and Signal Quality Algorithms for Sleep Apnea Detection and Monitoring. Sensors.

[38] Lan Z., Sourina O., Wang L., Scherer R., Muller-Putz G.R. (2019). Domain Adaptation Techniques for EEG-Based Emotion Recognition: A Comparative Study on Two Public Datasets. IEEE Trans. Cogn. Dev. Syst..

[39] Li X., Song D., Zhang P., Zhang Y., Hou Y., Hu B. (2018). Exploring EEG features in cross-subject emotion recognition. Front. Neurosci..

[40] Park J., Lee S., Jeon M. (2009). Atrial fibrillation detection by heart rate variability in Poincare plot. Biomed. Eng. Online.

[41] Natarajan A., Angarita G., Gaiser E., Malison R., Ganesan D., Marlin B.M. Domain adaptation methods for improving lab-to-field generalization of cocaine detection using wearable ECG. Proceedings of the 2016 ACM International Joint Conference on Pervasive and Ubiquitous Computing.

[42] Montes J., Young J.C., Tandy R., Navalta J.W. (2018). Reliability and Validation of the Hexoskin Wearable Bio-Collection Device During Walking Conditions. Int. J. Exerc. Sci..

[43] Wang J., Spicher N., Warnecke J., Haghi M., Schwartze J., Deserno T. (2021). Unobtrusive Health Monitoring in Private Spaces: The Smart Home. Sensors.

[44] Carre Technologies Inc (2008). (Hexoskin), Montreal, Canada. https://www.hexoskin.com/.

[45] (2010). Capical GmbH, Braunschweig, Germany. http://www.capical.de.

[46] Ganapathy N., Swaminathan R., Deserno T.M. (2021). Adaptive learning and cross training improves R-wave detection in ECG. Comput. Methods Programs Biomed..

